# Erenumab versus topiramate for the prevention of migraine – a randomised, double-blind, active-controlled phase 4 trial

**DOI:** 10.1177/03331024211053571

**Published:** 2021-11-07

**Authors:** Uwe Reuter, Marc Ehrlich, Astrid Gendolla, Axel Heinze, Jan Klatt, Shihua Wen, Peggy Hours-Zesiger, Jacqueline Nickisch, Christian Sieder, Christian Hentschke, Monika Maier-Peuschel

**Affiliations:** 1 Department of Neurology, Charité Universitätsmedizin Berlin, Berlin, Germany; 2 Universitätsmedizin Greifswald, Greifswald, Germany; 3 Novartis Pharma GmbH, Nuremberg, Germany; 4 Praxis Gendolla, Essen, Germany; 5 The Kiel Migraine and Headache Centre, Kiel, Germany; 6 Novartis Pharma AG, Basel, Switzerland; 7 Novartis AG, New York, NY, USA

**Keywords:** Erenumab, CGRP, migraine, topiramate, head-to-head study, prophylaxis

## Abstract

**Background:**

We compared the tolerability and efficacy of erenumab, a monoclonal antibody binding to the calcitonin gene-related peptide receptor, to topiramate for migraine prophylaxis in adults.

**Methods:**

HER-MES was a 24-week, randomised, double-blind, double-dummy, controlled trial conducted in 82 sites in Germany. Patients with ≥4 migraine days per month and naïve to study drugs were randomly assigned (1:1) to either subcutaneous erenumab (70 or 140 mg/month) plus topiramate placebo (erenumab group) or oral topiramate at the individual dose with optimal efficacy (50–100 mg/day) plus erenumab placebo (topiramate group).

The primary endpoint was medication discontinuation due to an adverse event during the double-blind phase. The proportion of patients that achieved ≥50% reduction from baseline in monthly migraine days during the last 3 months of the double-blind phase was a secondary endpoint.

**Results:**

Seven hundred and seventy-seven patients were randomised (from 22 February 2019 to 29 July, 2020) and 95.1% completed the study. In the erenumab group, 10.6% discontinued medication due to adverse events compared to 38.9% in the topiramate group (odds ratio, 0.19; 95% confidence interval 0.13–0.27; *p* < 0.001). Significantly more patients achieved a ≥50% reduction in monthly migraine days from baseline with erenumab (55.4% vs. 31.2%; odds ratio 2.76; 95% confidence interval 2.06–3.71; *p* < 0.001). No new safety signals occurred.

**Conclusions:**

Erenumab demonstrated a favourable tolerability and efficacy profile compared to topiramate.

Trial registration: ClinicalTrials.gov NCT03828539, URL: https://clinicaltrials.gov/ct2/show/NCT03828539

## Introduction

In migraine prophylaxis, tolerability is a major contributing factor to therapeutic success. Still, a recently published study shows that 28.2% of US migraine patients discontinue treatment within 6 months, mostly due to side effects (1).

Since evidence of comparative efficacy is limited, the therapeutic decisions are usually based on medication side effect profiles, patient characteristics and comorbidities (2,3). One of the most commonly used first-line therapy options in international guidelines is topiramate (2,3). Several placebo-controlled trials have demonstrated the efficacy of topiramate in migraine prevention (4–7), and it is considered to have the highest level of evidence to support its use as a migraine preventive treatment (2). A placebo-controlled study that included propranolol as an active control showed that topiramate at 100 mg/day is similarly effective to propranolol at 160 mg/day (7).

With erenumab, an antibody targeting the calcitonin gene-related peptide (CGRP) receptor, a new treatment option for migraine prophylaxis was introduced in 2018 (8,9), subsequently followed by other compounds targeting the CGRP protein. According to current guidelines and local reimbursement status, antibodies targeting the CGRP pathway are mainly considered an option for severely afflicted migraine patients who have failed or have not been suitable for at least two previous prophylactic treatments (2,10). So far, no study has been done in order to compare the efficacy of a monoclonal antibody targeting the CGRP pathway to that of a standard of care oral preventive drug.

HER-MES (Head-to-head study of erenumab against topiramate – Migraine study to assess tolerability and efficacy in a patient-centred setting) aimed to directly compare the tolerability and efficacy of the CGRP receptor antibody erenumab to topiramate. We report here the results of the 24-week double-blind, double-dummy trial, which included adult patients with at least 4 migraine days per month.

## Methods

### Study design

HER-MES was a randomised, double-blind, double-dummy, active-controlled, parallel-group phase 4 trial conducted at 82 study sites in Germany. The trial comprised a screening phase (up to 2 weeks) to assess eligibility, a baseline phase (4 weeks) to establish migraine day frequency and headache diary compliance, a double-blind, randomised treatment phase (24 weeks) and a safety follow-up phase (4 weeks). Study approval was granted by the State Office for Health and Social Affairs (Landesamt für Gesundheit und Soziales, Berlin, Germany). In addition, independent ethics committees at each trial centre approved the study protocol and its amendments. 

### Patients

Adults (18–65 years) with a history of migraine with or without aura for at least 12 months prior to screening who had never received treatment with topiramate or a monoclonal antibody targeting the CGRP pathway were enrolled. Migraine was defined according to the International Classification of Headache Disorders, 3rd edition (ICHD-3) (11). In the initial protocol, only patients with episodic migraine (4–14 monthly migraine days (MMDs) over the last 3 months prior to screening) were eligible. Patients also had to meet these criteria during a 4-week baseline phase, during which they had to record headache information daily in an electronic diary (eDiary). Study participation required ≥80% eDiary compliance during the baseline phase. In order to implement the recommendation of the health technology assessment (HTA) body to include a full migraine population in the trial, a protocol amendment permitted patients with chronic migraine to be enrolled. At this time, 43.8% of the total study population had been randomised, all of them patients with episodic migraine.

Patients were eligible if they had not received prior prophylactic migraine treatment (naïve) or, due to lack of efficacy or tolerability, had failed or had not been suitable for up to three previous prophylactic treatments from the following: Metoprolol/propranolol, amitriptyline, and flunarizine. Patients were excluded if they were older than 50 years at migraine onset, had a history of cluster headache or hemiplegic migraine, or were unable to differentiate migraine from other headaches. Patients were also excluded if they had previously received valproate or, in the event of chronic migraine, onabotulinumtoxin A, in line with recommendations of the German HTA bodies. The use of any medication for migraine prophylaxis within five half-lives, or a device or a procedure within 1 month prior to the start of the baseline phase and during the study, was prohibited. A complete list of the inclusion and exclusion criteria is provided in the appendix (pp. 3–5).

Patients were recruited under the supervision of study investigators at each site.

The study was conducted according to good clinical practice (GCP) guidelines. All patients were informed about study conduct, expected benefits, and potential risks, including possible adverse events (AEs) of both study drugs, and provided written consent to participate in the trial.

### Randomisation and masking

At the first visit in the double-blind treatment phase (DBTP), eligible patients were enrolled by investigators and randomly assigned in a 1:1 ratio to either the topiramate group (topiramate verum + erenumab placebo) or the erenumab group (erenumab verum + topiramate placebo). To account for the different doses of topiramate or erenumab we used matching placebo dummies; for topiramate, this included matching coloured placebo tablets. Randomisation was performed using interactive response technology (IRT) and stratification by MMD (4–7 days, 8–14 days, ≥15 days). Cenduit produced the randomisation list, provided the IRT and allocated the participants to the groups.

Novartis provided the investigational medicinal products in a double-dummy setting. The identity of treatments was concealed by use of study drug/matching placebo that were identical in packaging (i.e. identical blister packs and syringes), labelling, schedule of administration, appearance, taste, and odour.

Patients, investigator staff, persons performing the assessments, and Novartis personnel and their delegates remained blinded to the treatment identity from randomisation until conclusion of the primary analysis.

### Procedures

The 24-week DBTP comprised an up-titration phase of 6 weeks with weekly visits followed by a maintenance phase with visits at weeks 8, 12, 16, 20 and 24. At visits, topiramate or matching placebo was distributed in tablet blister packs for self-administration. During the 6-week up-titration phase, the intention was to increase dose weekly in 25 mg increments with the aim of reaching 100 mg/day according to the United States prescribing information (USPI) (12) and the European summary of product characteristics (SmpC) (13). To allow for a slower titration speed in line with clinical practice, patients could maintain a dose for longer than 1 week if deemed necessary. After 6 weeks, patients had to achieve a dose of at least 50 mg/day (according to the USPI (12) and European SmPC (13) the recommended dose is 100 mg/day; however, some patients can already benefit from 50 mg/day).

Erenumab or matching placebo was administered as subcutaneous injection every 4 weeks (±4 days) at the study site. The starting dose for erenumab was 70 mg (70 mg/ml per pre-filled syringe) or 140 mg (2 ×70 mg/ml) or equal amounts of matching placebo, based on investigator decision. If the starting dose was 70 mg erenumab/placebo and response was deemed insufficient, dose could be increased to 140 mg erenumab/placebo at any time throughout the treatment phase.

Dose reduction was not permitted for either drug at any point during the DBTP of the study. Upon discontinuation of medication, patients taking ≥75 mg topiramate/placebo had to taper off by reducing the dose by 50 mg per week.

Patients recorded information on duration and severity of migraine/non-migraine headache and intake of rescue medication in an eDiary daily during the baseline and the DBTP. Patient-recorded outcome questionnaires were to be completed either using the eDiary (medical outcome short form health survey version 2 [SF-36v2], headache impact test [HIT-6], treatment satisfaction questionnaire for medication [TSQM], Beck Depression Inventory [BDI-II]) or via automated telephone software (Columbia-suicide severity rating scale [C-SSRS]), according to the assessment schedule (see protocol in the appendix). Regular visits included physical and laboratory examinations, checks on vital signs, review of eDiary compliance, and documentation of concomitant medications and AEs. AEs were assessed at visits without the use of a checklist of common side effects.

### Outcomes

The primary endpoint was the proportion of patients who discontinued erenumab or topiramate medication due to an AE during the DBTP. The primary reason for medication discontinuation was assessed for each patient. Options included AEs, lack of efficacy, lost to follow up, patient or physician decision, and others. Patients who discontinued study medication during the DBTP could remain in the study provided that they completed their eDiary and visits. The secondary endpoint was the proportion of patients in each treatment group who achieved ≥50% reduction from baseline in MMD over months 4, 5 and 6 of the DBTP. A migraine day was defined according to IHS trial guidelines and in consistency with previous erenumab studies (14,15). A migraine attack was considered qualified when the headache lasted for at least 30 min with at least two of the following pain features: Unilateral, throbbing, moderate to severe intensity and exacerbation with physical activity and was accompanied by at least one of the following symptoms: Nausea, phono-/photophobia and vomiting.

Exploratory variables were the mean change in MMD from baseline over months 4, 5 and 6 and the patient reported outcomes SF-36v2 (16) and HIT-6 (16). The SF-36v2 is a multipurpose short-form survey to assess health-related quality of life. Its two component scores (the physical health component and the mental health component) are derived from eight subscales (physical functioning, physical role functioning, bodily pain, general health, vitality, social functioning, emotional role functioning, and mental health). A higher score corresponds to a more favourable health state (16). The HIT-6 is a six-item survey that assesses the adverse impact of headaches on social, role and cognitive functioning, vitality, and psychological distress. The final score is obtained by summation of the six items. A larger score reflects a greater adverse impact (17).

Safety was assessed by monitoring AEs, physical examination, measurement of vital signs, clinical laboratory assessments and electrocardiography.

### Statistical analysis

Based on results from previous studies with topiramate regarding discontinuation rates in patients with episodic migraine (9% for erenumab and 18% for topiramate (18)), it was estimated that a total of 700 patients with ≥4–15 migraine days would be needed to demonstrate superiority of erenumab with more than 90% power on a two-sided, 5% significance level. With the amendment to include patients with ≥15 migraine days per month, the calculated sample size was increased to 750 patients (375 per group) to account for the expected lower odds ratio regarding the discontinuation rate for topiramate in chronic migraine.

The full analysis set (FAS) and the safety analysis set (SAF) consisted of all randomised patients who received at least one dose of double-blind study medication. In the FAS, patients were analysed according to randomised treatment. In the SAF, patients were analysed based on the actual treatment received. No interim analysis was conducted.

The primary and secondary endpoints were analysed in the FAS with the use of a logistic regression model with the factor treatment and stratification factor (migraine days during the baseline phase). The odds ratio (OR) and its 95% confidence interval (CI) and *p*-values were calculated.

To reject the null hypotheses, odds of AE-related treatment discontinuation or achievement of ≥50% reduction from baseline were equal in both treatment groups (OR = 1). The null hypothesis was rejected if the two-sided *p*-value from the logistic regression model for the factor “treatment” was <0.05. However, superiority of erenumab for the endpoint “discontinuation” was claimed only if the direction was correct; that is, if the odds of response were lower under erenumab compared to topiramate. Additionally, estimates for the relative risk and the risk difference with 95% CI and *p*-value were calculated based on the Wald statistic.

For the primary endpoint, by definition, no missing values could occur.

For the secondary endpoint, subjects with missing eDiary entries in all months 4–6 visits were imputed as non-response. No subgroup analyses were planned.

Exploratory continuous efficacy outcomes were analysed in the FAS with a linear mixed effects model for repeated measures (MMRM), without missing value imputation.

Statistical analyses were done with SAS (version 9.2). A data monitoring committee was not required since both study drugs are on market.

## Results

**Figure 1.**
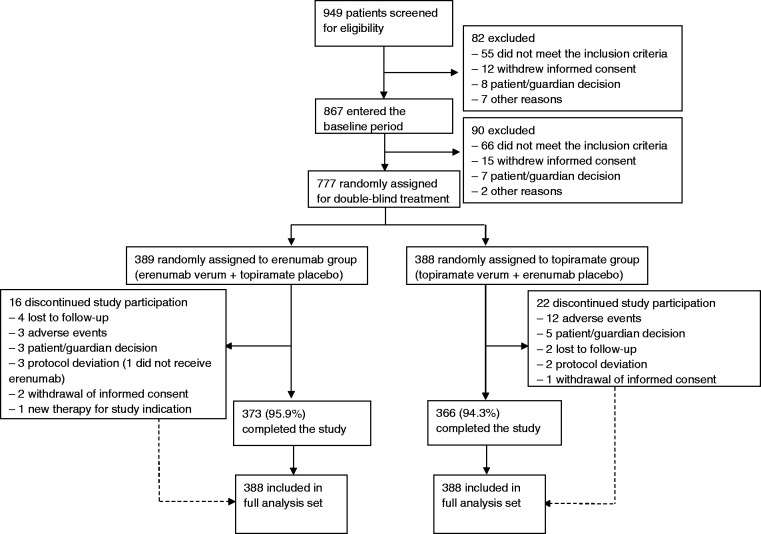
Study profile.

Between 22 February 2019 (first patient first visit), and 29 July 2020 (last patient last visit), 949 patients were screened for eligibility and 777 patients were randomised: 389 to the erenumab group and 388 to the topiramate group (Figure 1). One patient did not receive study medication and was excluded from the analysis, which led to identical FAS and SAF data sets; 739 (95.1%) patients completed the study until week 24. Among those, the mean eDiary compliance during the DBTP was 98.5% (SD 4.93).

Demographic and disease characteristics were well balanced between the groups (Table 1). At baseline, 64.7% of the total study population had 8–14 MMD. The mean number of MMD was 10.4 (3.9) days. 59.4% had never received prior preventive migraine medication.

**Table 1.** Baseline demographics and clinical characteristics.

	Erenumab (n = 388)	Topiramate (n = 388)	All patients (n = 776)
Age (years)	40.8 (12.4)	40.7(12.4)	40.7 (12.4)
range	18–66	18–65	18–66
Sex			
Women	331 (85.3%)	335 (86.3%)	666 (85.8%)
Men	57 (14.7%)	53 (13.7%)	110 (14.2%)
Ethnicity			
Caucasian	383 (98.7%)	387 (99.7%)	770 (99.2%)
Other	5 (1.3%)	1 (0.3%)	6 (0.8%)
Weight (kg)*	73.3 (17.9)	72.7 (17.5)	73.0 (17.7)
Body-mass index (kg/m²)*	25.6 (5.6)	25.3 (5.6)	25.5 (5.6)
Disease duration (years)	21.8 (12.5)	21.9 (12.4)	21.9 (12.4)
Migraine with aura^†^	131 (33.8%)	135 (34.8%)	266 (34.3%)
Acute headache medication use			
Migraine-specific	304 (78.4%)	320 (82.5%)	624 (80.4%)
Non-migraine-specific	74 (19.1%)	58 (14.9%)	132 (17.0%)
Prior prophylactic treatment attempts^‡^			
None (naïve)	232 (59.8%)	229 (59.0%)	461 (59.4%)
1 failed	115 (29.6%)	123 (31.7%)	238 (30.7%)
2 failed	37 (9.5%)	31 (8.0%)	68 (8.8%)
3 failed	4 (1.0%)	5 (1.3%)	9 (1.2%)
Baseline phase			
eDiary compliance ≥80%	385 (99.2%)	387 (99.7%)	772 (99.5%)
Monthly headache days^†^	11.4 (4.2)	11.5 (4.1)	11.5 (4.2)
Monthly migraine days^†^	10.3 (4.0)	10.5 (3.8)	10.4 (3.9)
4–7 monthly migraine days	94 (24.2%)	92 (23.7%)	186 (24.0%)
8–14 monthly migraine days	248 (63.9%)	254 (65.5%)	502 (64.7%)
≥15 monthly migraine days	43 (11.1%)	42 (10.8%)	85 (11.0%)
Beck Depression Inventory (BDI)-II total score severity grade			
Minimal depression (0–13)	371 (95.6%)	373 (96.1%)	744 (95.9%)
Mild depression (14–19)	17 (4.4%)	10 (2.6%)	27 (3.5%)
>19	0	5 (1.3%)	5 (0.6%)
HIT-6™ score^§^	63.6 (4.2)	63.9 (4.1)	63.8 (4.1)
SF-36v2 score			
Physical component	45.3 (7.1)	44.8 (7.2)	45.0 (7.2)
Mental component	51.5 (8.5)	52.1 (8.1)	51.8 (8.3)

Note: Data are mean (SD) or n (%) unless otherwise stated.

*n = 387 in the topiramate group.

^†^n = 387 in the erenumab group.

^‡^Out of propranolol/metoprolol, amitriptyline, flunarizine.

^§^n = 385 in the erenumab group, n = 384 in the topiramate group, data obtained at randomization visit.

^¶^n = 384 in the erenumab group, n = 381 in the topiramate group, data obtained at randomization visit.

In the erenumab group, 285 (73.5%) patients started with the 70 mg dose and for 165 (42.5%) patients, the dose was increased to 140 mg during the 24-week DBTP. A starting dose of 140 mg was chosen for 103 patients (26.5%). During months 4–6, 346 out of 388 patients (89.2%) were on active study medication.

In the topiramate group, all patients started with a dose of 25 mg/day. Detailed dose distribution during the 6-week up-titration phase is provided in Table S1 (appendix p. 6). At the end of week 6, 275 (70.9%) patients were still on medication. Among those, 207 (75.3%) achieved a daily dose of 100 mg topiramate, 49 (17.8%) received 75 mg and 19 (6.9%) received 50 mg daily, resulting in a mean topiramate dose of 92.1 mg/day. During months 4–6, 246 out of 388 patients (63.4%) were on active study medication.

**Figure 2.**
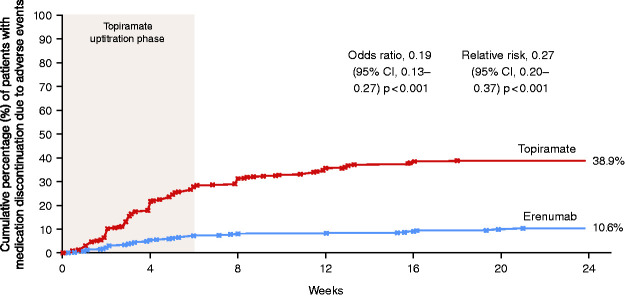
Cumulative percentage of patients who discontinued medication due to adverse events. Shading indicates the 6-week topiramate/placebo up-titration phase.

The proportion of patients who discontinued medication due to AEs during the 24-week DBTP in the erenumab group was 10.6% (41/388) versus 38.9% (151/388) in the topiramate group with an OR of 0.19 (95% CI 0.13–0.27, *p* < 0.001) and a relative risk (RR) of 0.27 (95% CI 0.20–0.37, *p* < 0.001) (Figure 2). At the end of week 6, 26.6% had already aborted medication in the topiramate group and 8.3% in the erenumab group (Figure 2). Over the course of the DBTP only one patient out of the total population who received erenumab terminated medication due to lack of efficacy. Patients that discontinued medication could remain in the study for further data collection.

In the last 3 months of the DBTP, 55.4% (215/388) of the patients in the erenumab group achieved a reduction in MMD of at least 50% from baseline (i.e. ≥50% responder rate), compared to 31.2% (121/388) in the topiramate group (OR 2.76; 95% CI 2.06–3.71; *p* < 0.001; RR 1.78; 95% CI 1.50–2.11; *p* < 0.001; Table 2). Patients in the erenumab group experienced a significantly greater reduction in mean MMD during months 4–6 than patients in the topiramate group (erenumab –5.86 versus topiramate –4.02, Δ −1.84, *p* < 0.001; Table 2).

**Table 2.** Efficacy over months 4 to 6 of the double-blind treatment phase and patient reported outcomes (FAS).

	Erenumab (n = 388)	Topiramate (n = 388)	OR/RR or difference (95% CI)	*p*-value
Secondary efficacy endpoint				
≥50% reduction from baseline in migraine days per month	215 (55.4%)	121 (31.2%)	OR 2.76 (2.06–3.71)RR 1.78 (1.50–2.11)	<0.001<0.001
Exploratory endpoints				
Monthly migraine days*	−5.86 (0.24)	−4.02 (0.24)	−1.84 (−2.43 to −1.25)	<0.001
HIT-6 (36–78)^†^	−10.9 (0.4)	−7.7 (0.4)	−3.2 (−4.3 to −2.1)	<0.001
SF-36v2 (0–100)^‡^				
Physical component	5.5 (0.4)	3.6 (0.4)	1.9 (1.0–2.8)	<0.001
Mental component	1.0 (0.5)	−1.2 (0.5)	2.2 (1.0–3.3)	<0.001

Note: Data are n (%) or adjusted mean change (SE) unless otherwise stated.

FAS = full analysis set.

*n = 383 in the erenumab group and n = 385 in the topiramate group.

^†^n = 379 in the erenumab group and n = 377 in the topiramate group.

^‡^n = 378 in the erenumab group and n = 374 in the topiramate group.

Furthermore, patients in the erenumab group reported a significantly larger reduction for both assessed quality of life questionnaires (HIT-6, SF-36v2).

Mean baseline values for HIT-6 at day one of the DBTP were reported at 63.6 in the erenumab group and 63.9 in the topiramate group, both fall into the highest level of the four HIT-6 categories attributing a severe impact by headache in this population (19). The reduction regarding the impact of headache on function was reported as −10.9 vs. −7.7; Δ −3.2; *p* < 0.001 for erenumab patients compared to patients in the topiramate group (Table 2). In correspondence, results obtained from the SF-36v2 questionnaire showed a significantly larger improvement in quality of life for patients in the erenumab group than for patients in the topiramate group. This was true for the physical component of this assay as well as for the mental component (Table 2).

Overall, study treatment-related AEs were more frequent in the topiramate group than in the erenumab group (81.2% vs. 55.4% of patients) (Table 3, and Table S2 in the appendix p. 7). In the topiramate group, the most frequent AEs that led to discontinuation of study medication were paraesthesia, disturbance in attention, fatigue, and nausea. In the erenumab group, these were fatigue, nausea, disturbance in attention and dizziness (Table 3).

**Table 3.** Adverse events reported during the double-blind treatment phase (SAF).

Event	Erenumab (n = 388)	Topiramate (n = 388)
Study treatment related adverse event*	215 (55.4%)	315 (81.2%)
Study treatment related serious adverse event	1 (0.3%)	2 (0.5%)
Adverse event leading to treatment discontinuation^†^^*^	41 (10.6%)	151 (38.9%)
Adverse event leading to treatment discontinuation reported by ≥2% of patient in any trial group		
Paraesthesia	0 (0.0%)	38 (9.8%)
Disturbance in attention	7 (1.8%)	36 (9.3%)
Fatigue	9 (2.3%)	29 (7.5%)
Nausea	8 (2.1%)	26 (6.7%)
Dizziness	4 (1.0%)	21 (5.4%)
Depression	3 (0.8%)	14 (3.6%)
Vertigo	4 (1.0%)	13 (3.4%)
Irritability	0 (0.0%)	10 (2.6%)
Dysgeusia	0 (0.0%)	10 (2.6%)
Mood swings	3 (0.8%)	9 (2.3%)
Depressed mood	1 (0.3%)	9 (2.3%)
Decreased appetite	1 (0.3%)	8 (2.1%)

Note: Data are number of patients (%).

SAF = safety analysis set.

*Study treatment related adverse events are detailed in Table S2 in the appendix, p. 7.

^†^Number of patients with at least one event leading to treatment discontinuation. One patient could report multiple adverse events leading to treatment discontinuation.

*The primary endpoint (adverse events leading to treatment discontinuation) was calculated from the FAS.

## Discussion

HER-MES is, to our knowledge, the first randomised controlled study that directly compares an antibody targeting the CGRP pathway to a standard of care prophylactic migraine treatment. It demonstrates that the tolerability of erenumab is superior to that of topiramate, and that patients in the erenumab treatment group had a significantly higher probability of achieving a clinically meaningful improvement in migraine frequency. This translated into a significantly reduced impact of headache on function and an improved quality of life with erenumab compared to topiramate.

Pivotal trials have demonstrated that both topiramate and erenumab are effective in migraine prevention (5–8,15,18,20–22). However, interventional, and observational studies have revealed that discontinuation rates are relatively high for topiramate (1,18,22,23). Tolerability is a prerequisite for an effective migraine drug to achieve meaningful improvement in a broad migraine population. Thus, our primary objective was to compare the tolerability of topiramate and erenumab measured as the rate of medication discontinuation due to AEs.

The main reasons that led to discontinuation of topiramate were paraesthesia, disturbance in attention and negative effects on mood. These are well described, typical side effects of topiramate (24,25). In the erenumab group, AEs that caused treatment discontinuation were dispersed. The most frequent AEs were fatigue, nausea, and disturbance in attention. None of them had occurred more often than placebo in previous placebo-controlled trials (8,15,20,21).

The discontinuation rates due to AEs in HER-MES were higher than in placebo-controlled studies in both treatment groups. We hypothesise that this can be largely attributed to a nocebo effect introduced by the rigorous double-dummy design. Nocebo describes a situation where a patient experiences AEs because he/she expects that a treatment will cause harm. An estimate for the frequency of nocebo is the proportion of patients that experience side effects under placebo treatment. A meta-analysis showed that more than 40% of the placebo-treated patients report side effects in preventive treatment for primary headache (26). The following observations support our hypothesis: First, the discontinuation rate for erenumab in HER-MES was similar to that for placebo in placebo-controlled studies of topiramate (18). Second, AEs that led to treatment discontinuation in the erenumab group in HER-MES were events that had not been reported more frequently than placebo in pivotal trials (8,15,20,21). Additionally, they included side effects that are typical for topiramate but are rather unspecific, such as disturbance of attention or fatigue. This may reflect the patients’ expectations of experiencing side effects of topiramate in both study groups.

Whereas previous studies in migraine preventive treatment do not take into account the impact of tolerability, the unique design of HER-MES for the first time conveys the real-world situation in a randomised-controlled design. Namely, efficacy endpoints were analysed in composite populations with patients on therapy and patients who had stopped medication but continued daily reporting. During the last 3 months of the DBTP, more patients were on erenumab therapy than on topiramate, which contributes to the better outcome. This underlines the importance of a good tolerability profile in achieving best possible results in migraine prevention.

Efficacy data obtained for erenumab was in line with the STRIVE trial (15). For topiramate, the 50% responder rates in HER-MES appear lower than in placebo-controlled trials (37% vs. 46.3% (17)). However, previous topiramate studies have used headache frequency instead of migraine days to calculate 50% responder rates, which limits comparison (18,24). Furthermore, the mean topiramate dose in the total group (composite population) was lower than in previous studies (18).

HER-MES includes a broad migraine population with 2/3 of the patients in the high-frequency migraine spectrum. Despite a mean disease duration of about 20 years, almost 60% of the patients had not received previous prophylactic treatment, which underlines the long-standing problem of undertreatment in migraine and may reflect the German healthcare situation. Many patients are reluctant to initiate a prophylactic therapy due to the poor tolerability of non-specific oral migraine preventive drugs. For the typical migraine patient, who is female and young to middle-aged, the cognitive and psychological side effects of topiramate can be especially disturbing in terms of occupational activities. This study demonstrates that compared to topiramate, erenumab substantially increases treatment acceptance. In combination with its good efficacy profile, it addresses the unmet need for a targeted and efficient treatment option that is well-tolerated in patients with episodic and chronic migraine.

A limitation of this study is the lack of a placebo group to judge nocebo and placebo effects. However, both erenumab and topiramate have been thoroughly tested and shown to be superior in efficacy against placebo. Since there were no questions open regarding the pharmacological effect of both drugs, we also decided against a third placebo group for ethical reasons.

Another potential weakness is partial unblinding due to typical side effects of topiramate. All patients were aware of the potential side effects of both study drugs in line with the GCP requirements for patient information before participation in a clinical trial. Patients who expected to experience side effects of topiramate might have been more prone to discontinue medication. To minimise this potential source of bias, the study was rigorously blinded in accordance with the recent guidelines for migraine trials (14) and employed an elaborate double-dummy design. Patients were not provided with a checklist to report AEs but rather reported them freely to site personnel at visits. We do not believe that unblinding is a major issue in HER-MES since the discontinuation rate was higher than in placebo-controlled trials for both study drugs. Additionally, AEs were in general more frequently reported for erenumab compared to placebo-controlled trials and included typical side effects of topiramate (e.g. paraesthesia). Since side effects typical for topiramate have not been reported as common for erenumab, either in placebo-controlled trials or in current public AE databases, this most likely indicates a nocebo effect as discussed above.

In HER-MES, patients could not reduce the dose of topiramate during the DBTP, although dose reduction of topiramate occurs in clinical practice. Down-titration was not permitted in order to comply with the treatment schemes specified in the current European and US summaries of product characteristics for topiramate (12,13). Consequently, the discontinuation rate might be higher than in the real-world situation. However, we implemented measures to reflect the clinical situation as closely as possible, and to avoid enforcing a high AE-related discontinuation rate under topiramate by enabling topiramate up-titration without strict weekly dose increments.

## Conclusions

Compared to topiramate, treatment with erenumab has a superior tolerability profile and a significantly higher efficacy. HER-MES supports the potential of erenumab in overcoming issues of low adherence in clinical practice observed with topiramate, lessening migraine burden and improving quality of life in a broad migraine population.

## Clinical implications

- First head-to-head comparison for a CGRP receptor antibody vs. a standard of care drug demonstrating superiority of erenumab vs. topiramate.
- Only a tolerable drug can be effective.
- Taking into account efficacy and tolerability data for every patient included in the trial (on-drug and drug discontinued) we tried to reflect real-world clinical conditions.
- With more trials showing similar results, treatment hierarchy in migraine prevention might change in the future.

## Supplemental Material

sj-pdf-1-cep-10.1177_03331024211053571 - Supplemental material for Erenumab versus topiramate for the prevention of migraine – a randomised, double-blind, active-controlled phase 4 trial Click here for additional data file. Supplemental material, sj-pdf-1-cep-10.1177_03331024211053571 for Erenumab versus topiramate for the prevention of migraine – a randomised, double-blind, active-controlled phase 4 trial by Uwe Reuter, Marc Ehrlich, Astrid Gendolla, Axel Heinze, Jan Klatt, Shihua Wen, Peggy Hours-Zesiger, Jacqueline Nickisch, Christian Sieder, Christian Hentschke and Monika Maier-Peuschel in Cephalalgia

sj-pdf-2-cep-10.1177_03331024211053571 - Supplemental material for Erenumab versus topiramate for the prevention of migraine – a randomised, double-blind, active-controlled phase 4 trial Click here for additional data file. Supplemental material, sj-pdf-2-cep-10.1177_03331024211053571 for Erenumab versus topiramate for the prevention of migraine – a randomised, double-blind, active-controlled phase 4 trial by Uwe Reuter, Marc Ehrlich, Astrid Gendolla, Axel Heinze, Jan Klatt, Shihua Wen, Peggy Hours-Zesiger, Jacqueline Nickisch, Christian Sieder, Christian Hentschke and Monika Maier-Peuschel in Cephalalgia

sj-pdf-3-cep-10.1177_03331024211053571 - Supplemental material for Erenumab versus topiramate for the prevention of migraine – a randomised, double-blind, active-controlled phase 4 trial Click here for additional data file. Supplemental material, sj-pdf-3-cep-10.1177_03331024211053571 for Erenumab versus topiramate for the prevention of migraine – a randomised, double-blind, active-controlled phase 4 trial by Uwe Reuter, Marc Ehrlich, Astrid Gendolla, Axel Heinze, Jan Klatt, Shihua Wen, Peggy Hours-Zesiger, Jacqueline Nickisch, Christian Sieder, Christian Hentschke and Monika Maier-Peuschel in Cephalalgia
