# Supplementary material for: Erenumab versus topiramate for the prevention of migraine – a randomised, double-blind, active-controlled phase 4 trial
Source: Cephalalgia. 2021 Nov 7;42(2):108–18. doi: 10.1177/03331024211053571 (PMC8793299; doi:10.1177/03331024211053571)
Supplement: sj-pdf-1-cep-10.1177_03331024211053571 - Supplemental material for Erenumab versus topiramate for the prevention of migraine – a randomised, double-blind, active-controlled phase 4 trial [file sj-pdf-1-cep-10.1177_03331024211053571.pdf]

Supplementary Table S2: Study treatment-related adverse events reported by ≥2% in any trial group (SAF)

Event	Erenumab (n = 388)	Topiramate (n = 388)
Paresthesia	17 (4• 4%)	155 (39• 9%)
Fatigue	38 (9• 8%)	67 (17• 3%)
Nausea	26 (6• 7%)	65 (16• 8%)
Disturbance in attention	18 (4• 6%)	63 (16• 2%)
Dizziness	20 (5• 2%)	51 (13• 1%)
Constipation	44 (11• 3%)	12 (3• 1%)
Decreased appetite	8 (2• 1%)	35 (9• 0%)
Taste disorder	0 (0• 0%)	24 (6• 2%)
Vertigo	17 (4• 4%)	23 (5• 9%)
Dysgeusia	3 (0• 8%)	23 (5• 9%)
Weight decreased	3 (0• 8%)	22 (5• 7%)
Dry mouth	8 (2• 1%)	18 (4• 6%)
Irritability	5 (1• 3%)	18 (4• 6%)
Mood swings	8 (2• 1%)	16 (4• 1%)
Diarrhea	7 (1• 8%)	16 (4• 1%)
Depression	6 (1• 5%)	16 (4• 1%)
Sleep disorder	16 (4• 1%)	6 (1• 5%)
Depressed mood	1 (0• 3%)	14 (3• 6%)
Hypoesthesia	2 (0• 5%)	13 (3• 4%)
Upper abdominal pain	11 (2• 8%)	10 (2• 6%)
Aphasia	2 (0• 5%)	11 (2• 8%)
Insomnia	6 (1• 5%)	10 (2• 6%)
Memory impairment	1 (0• 3%)	10 (2• 6%)
Dyspepsia	6 (1• 5%)	9 (2• 3%)
Dysesthesia	2 (0• 5%)	8 (2• 1%)
Headache	2 (0• 5%)	8 (2• 1%)

*Data are n (%). One patient could report multiple adverse events.
