# Supplementary material for: Erenumab versus topiramate for the prevention of migraine – a randomised, double-blind, active-controlled phase 4 trial
Source: Cephalalgia. 2021 Nov 7;42(2):108–18. doi: 10.1177/03331024211053571 (PMC8793299; doi:10.1177/03331024211053571)
Supplement: sj-pdf-2-cep-10.1177_03331024211053571 - Supplemental material for Erenumab versus topiramate for the prevention of migraine – a randomised, double-blind, active-controlled phase 4 trial [file sj-pdf-2-cep-10.1177_03331024211053571.pdf]

Supplementary Material – Inclusion and Exclusion Criteria

Inclusion criteria

Patients eligible for inclusion in this study must fulfill all of the following criteria. For inclusion purposes, onemonth equals one full calendar month.

During the Screening Epoch:

1. Patient is capable of understanding the nature, significance and implications of the clinical trial. Writteninformed consent must be obtained before any assessment is performed.

2. Adults ≥18 to ≤65 years of age upon entry into screening.

3. Documented history of migraine (with or without aura) for ≥12 months prior to screening according to theInternational Classification of Headache Disorders-3rd Edition (ICHD-3).

4. ≥4 migraine days per month (in at least two separate attacks) (based on ICHD-3 criteria) on average acrossthe last 3 fully completed calendar months prior to screening based on retrospective reporting.

(5. < 15 days of headache symptoms on average across the last 3 fully completed calendar

months)Criterion 5 was removed with the amendment to include patients with chronic migraine.

6. Patients must be either naïve or not suitable for or could have failed up to three prophylactic treatments outof: propranolol/metoprolol, amitriptyline, flunarizine.

The following definitions are applicable for inclusion criteria 6:

- Efficacy failure is defined as "no meaningful reduction in headache frequency after administration of the respective medication for an adequate period of time (at least 2 –3 months are recommended by the European Headache Federation treatment guidelines) at generally accepted therapeutic dose(s) based onthe investigator's assessment within the last 5 years prior to screening."
- Tolerability failure is defined as "documented discontinuation due to adverse events of the respectivemedication at any previous time."
- "Not suitable" for the purpose of this study is defined as "patient is not considered to be suitable forthe treatment for medical reasons such as contraindications or precautions included in local labels, national guidelines or other locally binding documents" as confirmed by the treating physician.

7. Patients on non-pharmacologic treatments (e.g., biofeedback, psychotherapy or other locally accepted andendorsed interventions for migraine) must have been stable on this treatment for at least 3 months prior to baseline.

During the Baseline Epoch:

8. Migraine frequency of ≥4 days during the Baseline Epoch, confirmed by the eDiary.

9. ≥80% eDiary compliance during the Baseline Epoch.

Exclusion criteria

Patients fulfilling any of the following criteria are not eligible for inclusion in this study. No additional exclusions may be applied by the investigator, in order to ensure that the study population will be representativeof all eligible patients. Calendar months are used for exclusion purposes.

1. Older than 50 years of age at migraine onset.

2. Unable to differentiate migraine from other headaches.

3. History of cluster headache or hemiplegic migraine headache.

4. Patients who have already been treated with topiramate, valproate or onabotulinumtoxin A.

5. Use of a prophylactic migraine medication within 5 half-lives, or a device or procedure within one monthprior to the start of the baseline phase or during the baseline phase.

6. Use of the following for any indication in the 1 month prior to the start of the baseline phase or during thebaseline phase: Opioid- or butalbital-containing analgesics ≥4 days/month.

7. Anticipated to require any excluded medication (see Section 5.5.8, Table 5-1), device or procedure (e.g.,occipital nerve stimulators, transcranial magnetic stimulation,) during the study.

8. Active chronic pain syndromes (e.g., fibromyalgia or chronic pelvic pain).

9. History or current evidence of major psychiatric disorder (such as schizophrenia, bipolar disorder or type Bpersonality disorder that might interfere with the ability to properly report clinical outcomes).

10. Evidence of drug or alcohol abuse or dependence within 12 months prior to screening, based on medicalrecords or patient self-report.

11. Current evidence of depression based on a BDI-II total score of >19 at screening. Patients with anxiety disorder and/or major depressive disorder are permitted in the study if they are considered by the investigator to be stable and are taking no more than one medication per disorder. Patients must have been on a stable dosewithin the 3 months prior to the start of the baseline phase.

12. History of seizure disorder or other significant neurological conditions other than migraine.

13. Score “yes” on item 4 or item 5 of the Suicidal Ideation section of the Columbia Suicide Severity RatingScale (C-SSRS), if this ideation occurred in the past 6 months, or “yes” on any item of the Suicidal Behaviorsection, except for the “Non-Suicidal Self-Injurious Behavior” (item also included in the Suicidal Behavior section), if this behavior occurred in the past 2 years.

14. Myocardial infarction, stroke, transient ischemic attack, unstable angina, or coronary artery bypass surgeryor other revascularization procedures within 12 months prior to screening.

15. History or current diagnosis of ECG abnormalities indicating significant risk of safety for patientsparticipating in the study.

16. History of malignancy of any organ system (other than localized basal cell carcinoma of the skin or in situcervical cancer), treated or untreated, within the past 5 years, regardless of whether there is evidence of local recurrence or metastases.

17. Hepatic disease by history or total bilirubin ≥2×ULN or ALT or AST ≥3xULN as assessed by centrallaboratory at initial screening.

18. Pregnant or nursing (lactating) women.

19. Women of child-bearing potential, defined as all women physiologically capable of becoming pregnant, unless they are using highly effective methods of contraception during dosing and for 110 days after stoppingof study medication. Highly effective contraception methods include:

- • Total abstinence (when this is in line with the preferred and usual lifestyle of the patient). Periodic abstinence (e.g., calendar, ovulation, symptothermal, post-ovulation methods) and withdrawal are notacceptable methods of contraception.
- • Female sterilization (have had surgical bilateral oophorectomy with or without hysterectomy) total hysterectomy or tubal ligation at least 6 weeks before taking investigational drug. In case of oophorectomyalone, only when the reproductive status of the woman has been confirmed by follow up hormone level assessment.
- • Male sterilization (at least 6 months prior to screening). For female patients on the study, the vasectomized male partner should be the sole partner for that patient and should have received medicalassessment of surgical success.
- • Use of oral, (estrogen and progesterone), injected or implanted hormonal methods of contraception or placement of an intrauterine device (IUD) or intrauterine system (IUS) or other forms of hormonal contraception that have comparable efficacy (failure rate <1%), e.g. hormone vaginal ring or transdermalhormone contraception.
- • In case of use of oral contraception women should have been stable on the same pill for a minimum of 3months before taking investigational drug.
- • Women are considered post-menopausal and not of child bearing potential if they have had 12 months ofnatural (spontaneous) amenorrhea with an appropriate clinical profile (e.g. age appropriate, history of vasomotor symptoms) or have had surgical bilateral oophorectomy (with or without hysterectomy), total hysterectomy or tubal ligation at least 6 weeks ago. In the case of oophorectomy alone, only when the reproductive status of the woman has been confirmed by follow up hormone level assessment is she considered not of child bearing potential.

20. Use of other investigational drugs within 5 half-lives of enrollment, or until the expected pharmacodynamiceffect has returned to baseline, whichever is longer.

21. History of hypersensitivity to the study drug or its excipients (including topiramate) or latex.

22. Patients not suitable for treatment with topiramate according to topiramate SmpC. Conditions listed under 4.4 Warnings and precautions in topiramate SmPC (e.g., risk of/history of nephrolithiasis, decreased renal function, risk of/history of eye disorders, risk of/history of metabolic acidosis) could be deemed as exclusioncriterion at discretion of the investigator.

23. Any prior exposure to (investigational) prophylactic migraine products targeting the CGRP pathway,including previous erenumab studies.

24. Unlikely to be able to complete all protocol required study visits or procedures, and/or to comply with allrequired study procedures (e.g., independent completion of electronic diary items) to the best of the patient'sand investigator's knowledge.

25. Patients who may be dependent on the sponsor or investigator.

26. Patient has not been committed to an institution by virtue of an order issued either by the judicial or theadministrative authorities.
