# Supplementary material for: Erenumab versus topiramate for the prevention of migraine – a randomised, double-blind, active-controlled phase 4 trial
Source: Cephalalgia. 2021 Nov 7;42(2):108–18. doi: 10.1177/03331024211053571 (PMC8793299; doi:10.1177/03331024211053571)
Supplement: sj-pdf-3-cep-10.1177_03331024211053571 - Supplemental material for Erenumab versus topiramate for the prevention of migraine – a randomised, double-blind, active-controlled phase 4 trial [file sj-pdf-3-cep-10.1177_03331024211053571.pdf]

Supplementary Table S1: Dose distribution for topiramate/placebo during the first 6 weeks of the double-blind treatmentphase (topiramate uptitration phase)

	**Erenumab group (Topiramate placebo) (N=388)**	**Topiramate group (Topiramate verum) (N=388)**
**Dose**		
Day 1		
25 mg	387 (100• 0%)	388 (100• 0%)
50 mg	0	0
75 mg	0	0
100 mg	0	0
missing	1	0
Week 1		
25 mg	25 (6• 6%)	44 (11• 8%)
50 mg	355 (93• 4%)	330 (88• 2%)
75 mg	0	0
100 mg	0	0
missing	8	14
Week 2		
25 mg	6 (1• 6%)	11 (3• 2%)
50 mg	59 (15• 9%)	98 (28• 3%)
75 mg	305 (82• 4%)	237 (68• 5%)
100 mg	0	0
missing	18	42
Week 3		
25 mg	2 (0• 5%)	3 (0• 9%)
50 mg	30 (8• 2%)	49 (15• 1%)
75 mg	87 (23• 8%)	109 (33• 6%)
100 mg	246 (67• 4%)	163 (50• 3%)
missing	23	64
Week 4		
25 mg	0	2 (0• 7%)
50 mg	14 (3• 9%)	24 (8• 0%)
75 mg	50 (13• 9%)	71 (23• 6%)
100 mg	296 (82• 2%)	204 (67• 8%)
missing	28	87
Week 5		
25 mg	0	0
50 mg	9 (2• 5%)	21 (7• 4%)
75 mg	35 (9• 8%)	52 (18• 2%)
100 mg	313 (87• 7%)	212 (74• 4%)
missing	31	103
Week 6		
25 mg	0	0
50 mg	8 (2• 3%)	19 (6• 9%)
75 mg	35 (9• 9%)	49 (17• 8%)
100 mg	310 (87• 8%)	207 (75• 3%)
missing	35	113
